# Chronic Fatigue Syndrome: A Case Report Highlighting Diagnosing and Treatment Challenges and the Possibility of Jarisch–Herxheimer Reactions If High Infectious Loads Are Present

**DOI:** 10.3390/healthcare9111537

**Published:** 2021-11-10

**Authors:** Rachel K. Straub, Christopher M. Powers

**Affiliations:** Division of Biokinesiology and Physical Therapy, University of Southern California, 1540 E. Alcazar St., CHP-155, Los Angeles, CA 90089-9006, USA; powers@pt.usc.edu

**Keywords:** myalgic encephalomyelitis (ME), chronic fatigue syndrome (CFS), post-exertional malaise, die-off reactions, chronic illness, Lyme disease, Epstein–Barr virus, Mycoplasma pneumonia, candida, orthostatic intolerance, light therapy, eye movement desensitization and reprocessing (EMDR), emotional freedom technique (EFT)

## Abstract

Myalgic encephalomyelitis/chronic fatigue syndrome (ME/CFS) is a complex multi-system disease with no cure and no FDA-approved treatment. Approximately 25% of patients are house or bedbound, and some are so severe in function that they require tube-feeding and are unable to tolerate light, sound, and human touch. The overall goal of this case report was to (1) describe how past events (e.g., chronic sinusitis, amenorrhea, tick bites, congenital neutropenia, psychogenic polydipsia, food intolerances, and hypothyroidism) may have contributed to the development of severe ME/CFS in a single patient, and (2) the extensive medical interventions that the patient has pursued in an attempt to recover, which enabled her to return to graduate school after becoming bedridden with ME/CFS 4.5 years prior. This paper aims to increase awareness of the harsh reality of ME/CFS and the potential complications following initiation of any level of intervention, some of which may be necessary for long-term healing. Treatments may induce severe paradoxical reactions (Jarisch–Herxheimer reaction) if high infectious loads are present. It is our hope that sharing this case will improve research and treatment options for ME/CFS.

## 1. Background

Myalgic encephalomyelitis/chronic fatigue syndrome (ME/CFS) is a debilitating and life-altering disease that affects people around the world, including as many as 2.5 million Americans [1]. Clinically, ME/CFS manifests as debilitating fatigue that worsens with physical or mental activity that is not relieved by rest and is not caused by excessive exertion [1]. Because of the severe impact of ME/CFS on general function (both mental and physical) and no accepted treatment, approximately 25% of patients are house or bedbound [1]. Some are so severe in function that they require tube-feeding and are unable to tolerate light, sound, and human touch [2,3].

Diagnosing ME/CFS remains a challenge, and it has been estimated that approximately 85% of patients remain undiagnosed [1]. In addition, information about ME/CFS is not taught in the majority of the nations’ medical schools [1], which has contributed to widespread disbelief and uncertainty among health care providers, many who do not accept ME/CFS as a genuine clinical entity [1,4,5]. As such, patients are frequently misdiagnosed with a psychological condition [1,4,5]. 

For those diagnosed with ME/CFS, long-term prognosis remains poor. Patients with ME/CFS have a lower quality of life compared to patients with other chronic diseases, such as cancer, multiple sclerosis, and stroke [6,7,8]. In addition, little progress has been made on developing diagnostics and treatments for ME/CFS in recent decades. Currently, there is no cure and no FDA-approved treatment for ME/CFS. ME/CFS is the most underfunded disease relative to disease burden among all the diseases funded by the United States National Institutes of Health (NIH) [8]. Although studies have documented a wide range of abnormalities in patients with ME/CFS (e.g., central and autonomic nervous systems, metabolic dysfunctions, compromised immunity, and chronic infections), most patients with ME/CFS have “normal” standard lab tests, making a definitive diagnosis difficult [9,10,11]. 

In this case report, we describe the complex medical history of one severe ME/CFS patient and her efforts to recover over a 4-year period, which resulted in her return to graduate school. The overall goal of this case report was to (1) describe how past events may have contributed to the development of severe ME/CFS in a single patient, and (2) the extensive medical interventions that the patient has pursued in an attempt to recover, which enabled her to return to graduate school after becoming bedridden with ME/CFS 4.5 years prior. This paper aims to increase awareness of the harsh reality of ME/CFS and the potential complications following initiation of any level of intervention, some of which may be necessary for long-term healing. It is our hope that sharing this case will improve research and treatment options for ME/CFS. 

## 2. Case Presentation

Upon presentation to the rheumatologist’s office (August 2013), the patient’s vitals were normal [BMI: 21.1, BP: 95/47, pulse: 66, RR: 13, Temperature: 97.8°]. She was a graduate student (age 28 and Caucasian) but had been on medical leave since January 2013 (7 months). She had to move home with her parents because she was no longer capable of caring for herself. Previously an avid exerciser (running and weight training), she was no longer capable of any physical activity. Simple activities such as showering would force her back to bed (post-exertional malaise [1,12,13]) (Table 1). She reported that she was no longer capable of eating most foods, as her digestive tract felt like it was shutting down. Her diet consisted of easily digestible foods proposed to be helpful in treating chronic diseases [14]. She also reported taking a wide range of supplements (~15 total), including probiotics, zinc, vitamin D, and fish oil.

**Figure 1:**
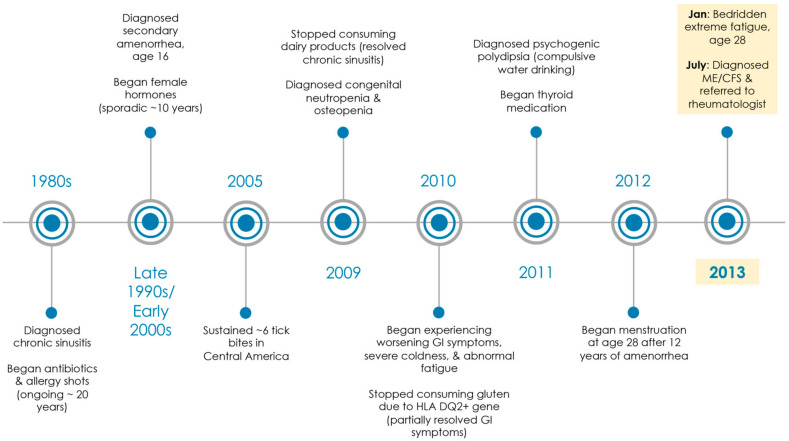
Medical history timeline. Patient saw over 20 medical specialists (i.e., pediatrics, allergy and immunology, gynecology, gastroenterology, endocrinology, hematology, infectious disease, internal medicine, alternative medicine, urgent care, and family medicine) from 1980s through 2013. In 2013, she was diagnosed with ME/CFS at age 28 by a family medicine physician and referred to a rheumatologist (MD, PhD).

From January 2013 to July 2013, the patient had been seen by four physicians with a complaint of profound fatigue and general malaise (urgent care, internal medicine, infectious disease, and family medicine). The most recent family medicine physician diagnosed her with ME/CFS in July 2013 (Figure 1) based on recognized criteria from the Centers for Disease Control and Prevention (CDC), which included profound disabling fatigue for at least 6 months that remained unexplained and was accompanied by frequent sore throats, impaired cognitive function, post-exertional malaise (Table 1) [1,12,13], unrefreshing sleep, headaches, and joint/muscle pain [15]. The patient also met the definition for ME/CFS as described by the ME International Consensus Criteria (ICC) based on energy production/transportation impairments (e.g., thermostatic instability and dizziness), immune/gastrointestinal/genitourinary impairment (e.g., chronic flu-like symptoms that worsened with exertion), neurological impairment (e.g., cognitive dysfunction and unrefreshing sleep), and post-exertional neuroimmune exhaustion (e.g., post-exertional malaise) [16]. The patient was then referred to the present physician (rheumatologist with a PhD in immunology) for further workup and treatment. The family medicine physician was concerned by a positive test for Antinuclear Antibody (ANA) (suggesting a possible autoimmune disorder). In addition, the family medicine physician viewed recent lab tests as inconclusive for Epstein–Barr virus, Mycoplasma pneumonia, and Chlamydia pneumonia. 

## 3. Past Medical History

The patient had been struggling medically since early childhood and had seen over 20 medical specialists (Figure 1). She had a medical history of chronic sinusitis with no food allergies, amenorrhea, osteopenia, gluten intolerance (HLA DQ2+ gene), congenital neutropenia, polyuria, polydipsia, and hypothyroidism.

For chronic sinusitis, the patient had a 20+ history of long-term antibiotics, steroids, and allergy shots. She reported that despite these treatments, she spent most of her childhood chronically sick and was frequently absent from school. Her chronic sinusitis resolved in her early 20s once she stopped consuming dairy products. 

The patient was diagnosed in her 20s with congenital neutropenia by a hematologist and psychogenic polydipsia (compulsive water drinking) by an endocrinologist. In addition, she had a 10+ year history of amenorrhea until recently and had seen over 10 doctors of various specialties for this condition alone (including 5 different gynecologists). She had 1–2 instances of spotting at age 16 and no menses thereafter. All lab tests were reported normal (including an MRI of her pituitary and an ultrasound of her ovaries). She reported that she was on female hormones for at least 10 years, but she was not compliant as she suffered ongoing negative side effects (including migraines). Apparently, all 3 conditions (polydipsia/polyuria, neutropenia, and amenorrhea) were resolved after she was prescribed Amour Thyroid at age 28 for hypothyroidism despite “normal” labs. 

The patient reported being bitten by several ticks in Central America 8 years ago. When she returned home, she began experiencing flu-like symptoms. However, she has never tested positive for Lyme disease nor displayed a bullseye rash. The patient also described that she has had declining health over the past 6 years, but she was semi-stable until recently. During the fall of 2012, the patient was continuously fighting ongoing infections and had several bouts of the flu. She was working on a limited basis while going to school, but she had to quit work completely. She no longer had the capacity to exercise, and minimal time away from home would force her to bed for several hours. 

## 4. Differential Diagnosis

A physical exam was generally normal, except for yellow hyperpigmentation of the palms, dry eyes, and dry mouth. A salivary gland ultrasound indicated enlarged intraparenchymal lymph nodes with increased cortex to hilum ratio (right and left parotid glands) and multiple hypoechoic intraparenchymal areas (right and left submandibular glands). Based on the examination, the patient was diagnosed with Sicca syndrome (dry eyes/mouth). An unspecified disease of the salivary glands (high probability of Sjogren’s syndrome, an autoimmune disease) and unspecified inflammatory spondylopathy also were suspected. The patient’s medical history suggested that hypothyroidism needed to be addressed, along with possible causes for ME/CFS (including a reactive post-infectious process due to Mycoplasma pneumonia/Chlamydia pneumonia infection). Gluten intolerance (HLA DQ2+) was also noted. Laboratory serology tests ordered are presented in Table 2. 

The patient’s labs indicated low T4 (55.03 nmol/L, normal: 60–120), low serum iron (36 ug/dL, normal: 37–160), low free lambda chains (5.41 mg/L, normal: 5.71–26.3) with increased kappa to lambda ratio (2.78, normal: 0.26–1.65), a negative extractable nuclear antigen (ENA) panel, elevated ammonia (47 umol/L, normal: 11–35), borderline elevated Mycoplasma pneumonia IgG (193 U/mL, indeterminate: 100–320), borderline elevated Chlamydia trachomatis IgM (0.8, borderline: 0.8–1.0), and elevated Chlamydia pneumonia IgG (1:128, negative: <1:16). The patient was considered to be suffering from a post-infectious process due to Chlamydia pneumonia. IgG titers can be elevated from past exposure, as opposed to a post-infectious process [28,29]. A repeat sample drawn weeks later that demonstrated a significant rise of IgG titers would provide increased evidence of a post-infectious process [28,29]. Although Mycoplasma pneumonia and Chlamydia pneumonia (bacterial infections) are usually self-limiting, clinical manifestations can range from self-limiting to life-threatening, from pulmonary to extrapulmonary [28,30]. In addition, intestinal Candida was suspected based on elevated ammonia levels and the patient’s medical history of long-term antibiotics. 

## 5. Treatment 

**Figure 2:**
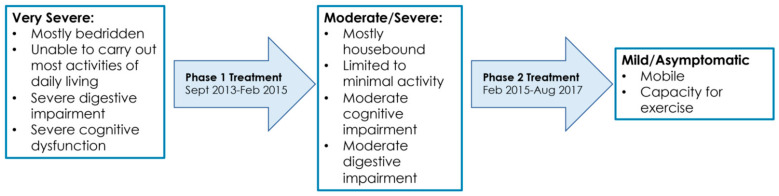
Symptom timeline over the course of treatment for ME/CFS (September 2013–August 2017).

For hypothyroidism, the patient was placed on Levothyroxine and was told to continue Armour Thyroid. For possible chronic bacterial infections, she was placed on antibiotics (doxycycline). She was also placed on Nystatin to combat possible Candida (fungal) overgrowth. She was expected to begin feeling better within 2–3 months of treatment, but the treating physician warned her that she may get worst before she gets better due to the Jarisch–Herxheimer reaction (die-off). The Jarisch–Herxheimer reaction is the worsening of existing symptoms (and the appearance of new symptoms) following treatment of several infectious diseases (including viral, bacterial, and fungal). It is an immunologic response that should not be confused with a medication allergy or an adverse reaction to treatment (Table 1) [17,18,19,20,21,22,23,24,25,26]. Authors have distinguished a Jarisch–Herxheimer reaction from a drug allergy based on resolution of symptoms despite continuation of therapy [21] and absence of liver test abnormalities [26]. An overview of the symptom changes observed over the course of treatment is shown in Figure 2. During treatment, it was not uncommon for the patient to revert backwards for days, weeks, or months at a time due to Jarisch–Herxheimer reactions. Any perturbation (e.g., new medication, dose increase, and reintroduction of past medication) would induce a Jarisch–Herxheimer reaction. During such periods, the patient’s tolerance to any of level of exertion (post-exertional malaise; Table 1) also decreased. 

## 6. Outcome and Follow-Up

### 6.1. September 2013 to February 2015

**Figure 3:**
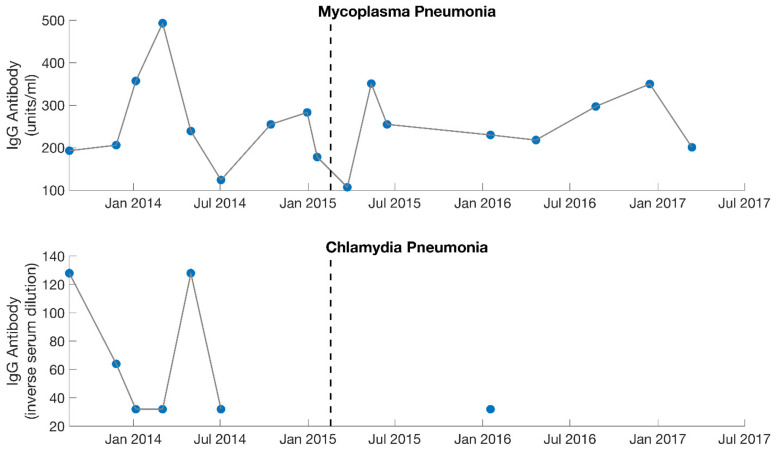
Serology results from IgG specific antibody levels for both Mycoplasma pneumonia and Chlamydia pneumonia. Dotted lines separate the 2 different phases of treatment (September 2013–February 2015, February 2015–August 2017). With the initiation of treatment, IgG levels rose for Mycoplasma pneumonia but fell for Chlamydia pneumonia. Mycoplasma pneumonia was viewed by the treating physician (MD, PhD) as the dominant contributing factor to the patient’s illness during the first phase of treatment. Large gaps are present for Chlamydia pneumonia as this was not measured consistently.

The patient was seen every 3–6 weeks over the course of 1.5 years. Prior to her first follow-up visit, she reported symptoms characteristic of Jarisch–Herxheimer reactions (severe joint and musculoskeletal pain, worsening fatigue, worsening cognitive function, migraines, drops in blood pressure, etc.) that she was struggling to control (Table 1). Based on this information, the doxycycline was discontinued. At her first follow-up, she was changed to a different antibiotic and was recommended various herbs for detoxification support (such as succinic acid, N-acetyl cysteine, bromelain, and a liver detox blend). Follow-up labs indicated that with the initiation of treatment, IgG titers rose for Mycoplasma pneumonia but fell for Chlamydia pneumonia (Figure 3). Therefore, Mycoplasma pneumonia was viewed by the treating physician as the more probable factor contributing to ME/CFS. In addition, the Jarisch–Herxheimer reactions she continued to experience were further suggestive of a chronic infection (Table 1) [17,18,19,20,21,22,23,24,25,26].

**Figure 4:**
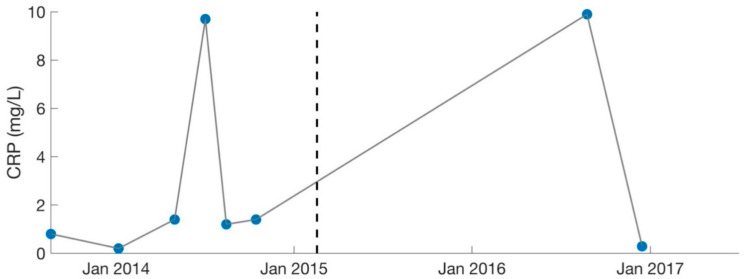
Serology results for CRP (inflammatory marker). Dotted lines separate the two different phases of treatment (September 2013–February 2015, February 2015–August 2017). CRP levels were deemed too high by the treating physician (MD, PhD) in July 2014 and August 2016. Large gaps are present as CRP was not measured consistently. C-Reactive Protein: CRP.

Over the course of 1.5 years, the patient was treated primarily for Mycoplasma pneumonia and was cycled among various antibiotics (including nebulized Gentamycin), in addition to synergists (such as Hydroxychloroquine and Dipyridamole [31]) to enhance antibiotic potency. Immune modulators (such as Colostrum, Astragalus, Andrographis, and Cordyceps) were also recommended to strengthen the patient’s immune system. Because of increasing ammonia levels (49 umol/L, normal: 11–35), the patient was changed to Fluconazole (antifungal) in December 2013 to treat suspected Candida. Her labs worsened in certain areas as treatment progressed, which was likely due to high levels of Jarisch–Herxheimer reactions [20,26,32]. C-Reactive Protein (CRP) reached a high of 9.7 mg/L in July 2014 (Figure 4). 

Low iodine (October 2014; 37.1 ug/L, normal: 40–92) and high cortisol levels (January 2014; 27.8 ug/dL, normal: 2.3–19.4) were also identified, so Kelp and adaptogen herbs (such as Ashwaganda and Eleutherococcus) were advised. The patient’s thyroid medication (Armour Thyroid and Levothyroxine) was also increased over the course of several months due to low T4 and/or low T3 levels. During the Spring of 2014 (~6 months after onset of treatment), she had improved enough to take a college course 1 day/week. However, she was not well enough to drive. In addition, too much mental exertion would cause her to crash to bed (post-exertional malaise [1,12,13]) (Table 1). She began to struggle with severe orthostatic intolerance and hypotension. Low aldosterone (which helps regulate blood pressure) was contributory, based on non-detectable blood levels. The herb Licorice Root was advised. When this alone was not adequate, Fludrocortisone was prescribed. By February 2015, she was still predominantly housebound, and although she was doing better cognitively, simple activity like walking was still too difficult on most days. Her overseeing physician at this point deemed her a mystery patient. Mycoplasma pneumonia levels appeared to be stabilizing (Figure 3) and frequent labs were not capable of identifying any additional abnormalities or infections. In addition, she remained abnormally sensitive to any level of treatment. An overview of the medications prescribed during phase 1 of treatment (September 2013 to February 2015) is provided in Table 3.

### 6.2. February 2015 to August 2017

To improve the chances of recovery, the patient made three major shifts in her treatment plan: (1) she began seeing other medical professionals and exploring non-traditional therapies. Most importantly, she began seeing a PhD clinical psychologist with expertise in energy psychology and various non-invasive techniques, such as eye movement desensitization and reprocessing (EMDR) and emotional freedom technique (EFT); (2) she started light therapy; and (3) she shifted her medication protocol from dominantly antibiotics to dominantly herbs.

EFT is a non-invasive method that involves purely tapping on various acupressure points and stating (or thinking) specific statements, while EMDR is a non-invasive method that involves simply moving the eyes in specific patterns while stating (or thinking) specific statements. Systematic reviews and meta-analyses have shown that EFT and EMDR are both effective for the treatment of depression [33,34], post-traumatic stress disorder (PTSD) [34,35], and anxiety [34,36]. In addition, both EFT and EMDR have been shown to improve chronic pain [37,38]. EFT also improves multiple physiological markers of health (such as blood pressure and cortisol) [37].

The patient saw the clinical psychologist approximately 1 day/week and spent 25–100% of her 1-h session on EFT and EMDR, with the focus on various topics related to her specific situation, such as improving health and well-being, clearing toxins and inflammation, and killing specific pathogens. Unfortunately, the patient experienced severe discomfort during her treatment sessions, including joint pain, headaches, excessive yawning, and flu-like symptoms. It would often take her several days to stabilize. Though the psychologist would frequently recommend that his patients perform EFT/EMDR at home (several times a day), the current patient was not capable.

In addition, light therapy was initiated. The patient purchased a LED face light with seven different colors for personal usage and began using it off label, all over her body (most frequently on top of her head). She used all seven colors, but the dominant colors were blue, red, and green. Blue light is effective in treating antibiotic-resistant strains of bacteria [39,40], in addition to acne (where blue light has FDA approval) [41,42]. Furthermore, red light therapy has been reported as a potential neuroprotective treatment for both Alzheimer’s and Parkinson’s patients [43], which has led to the emergence of red light bucket hats as a potential treatment for those with Parkinson’s disease [44]. Moreover, green light therapy has been shown to have anti-inflammatory effects in animal models [45] and decrease pain and improve quality of life in Fibromyalgia patients [46]. When the patient commenced treatment (initially with blue light on the face), she was not able to tolerate it more than 1–2 min without developing severe migraines. In time, light therapy became a daily crucial treatment (up to 6 h/day). 

Lastly, the patient shifted her medication protocol from dominantly antibiotics to dominantly herbs and began treating other possible infections (despite the lack of positive test results). She saw the overseeing physician (rheumatologist) every 1–3 months. The focus was no longer on Mycoplasma pneumonia, though this was still monitored (Figure 3). The overseeing physician recommended herbs for other possible conditions and infections for the patient to try on a trial basis, which included herbs for Epstein–Barr virus (Inosine, PABA, DMAE) and Lyme disease (Cat’s Claw). Broad-spectrum antimicrobials (such as Silver Hydrosol, Olive Leaf Extract, Anantamul, and Neem) were also recommended. Not surprisingly, the patient experienced severe reactions from all supplements. Therefore, the process of introducing new herbs (or increasing dosages of old ones) was performed with extreme care. Extensive labs were performed regularly, and although no novel infections were identified, inflammatory markers were sometimes elevated. CRP reached a high of 9.9 mg/L in August 2016 (Figure 4), which was during the period the patient was on an incremental light therapy protocol. The abnormal reactions to all forms of treatment (including non-invasive therapies described above) were suggestive of Jarisch–Herxheimer reactions [17,18,19,20,21,22,23,24,25,26] (Table 1). An overview of the medications prescribed during phase 2 of treatment (February 2015 to August 2017) is provided in Table 4.

### 6.3. August 2017

After 2.5 years of a revised treatment protocol, the changes made in the treatment plan were considered successful. In August 2017, the patient was well enough to move out from her parents’ home and resume graduate school, after becoming bedridden with ME/CFS 4.5 years prior. Prior to returning to graduate school in August 2017, the patient was stable for at least 6 months. Her diet had also expanded considerably for several months, and other than dairy and gluten, she had no restrictions. She was even back at the gym by April 2017 and was going for walks daily (something she was not capable of doing in over 4 years). She resumed graduate school with a very light schedule and maintenance protocol comprised of prescription medications (Armour Thyroid and Levothyroxine), extensive herbs (e.g., antimicrobials, antivirals, and immune modulators), and non-invasive therapies (EFT, EMDR, and light). A summary of the treatments used by the patient from September 2013 to August 2017 is provided in Table 5.

## 7. Discussion

The purpose of this case report was to describe the complex medical history of one severe ME/CFS patient and her efforts to recover over 4 years, which enabled her to return to graduate school. The overall goal of this case report was to (1) describe how past events may have contributed to the development of severe ME/CFS in a single patient, and (2) the extensive medical interventions that the patient has pursued in an attempt to recover. This paper aims to increase awareness of the harsh reality of ME/CFS and the potential complications following initiation of any level of intervention, some of which may be necessary for long-term healing. It is our hope that sharing this case will improve research and treatment options for ME/CFS. 

Given the patient’s complex case history (e.g., chronic sinusitis, amenorrhea, tick bites, congenital neutropenia, psychogenic polydipsia, and hypothyroidism), it is possible that these events contributed to her onset of severe ME/CFS in her late 20s. The fact that she began menstruation at age 28 after 12 years of amenorrhea (as noted above, the patient had only 1–2 instances of spotting at age 16 and no menses thereafter) with administration of thyroid hormones implies she had serious, long-term hypothyroidism (as absence of menarche by age 15 is statistically uncommon) [47]. Hypothyroidism can result in a wide range of medical problems that were reported by this female patient, including amenorrhea [48], compromised immune function (including neutropenia) [49,50], and severe sensitivity to cold [51]. In addition, thyroid hormones are necessary for normal kidney function [52], which could explain the resolution of polydipsia following administration. It is possible that 10 years of treatment with female hormones was unnecessary, which explains why the patient reported low compliance due to ongoing negative side effects. Given that administration of thyroid hormones also resolved neutropenia, polyuria, and polydipsia suggests that the patient was also misdiagnosed with psychogenic polydipsia and congenital neutropenia. The fact that the severity of her hypothyroidism negatively affected so many different organ systems likely made her more susceptible to ME/CFS. 

The patient’s 20+ year struggle with chronic sinusitis may have also been implicated in the patient’s development of ME/CFS. Although she was on long-term antibiotics, she reported she was still chronically sick. Therefore, it was logical that something else was contributory (dairy intolerance as noted in medical history). Adverse reactions to food can be the result of an immune-mediated reaction (i.e., food allergy) or non-immune reaction (i.e., food intolerance) [53]. The gold standard for the diagnosis of a food intolerance is a food challenge with the suspect food after elimination for several weeks [54,55]. Unfortunately, diagnostic tools available for suspected food allergies cannot accurately predict food intolerances [55], which explains why it took the patient decades to discover a dairy intolerance (as she had no food allergies). True food allergies typically occur within minutes to hours after exposure, while food intolerances typically occur hours to days after exposure [56]. 

The fact that the patient was treated with long-term antibiotics for decades for chronic sinusitis may have contributed to Candida overgrowth [57,58,59,60]. Some authors have called Candida: “a disease of antibiotics” [57]. Although the presence of Candida organisms is generally benign, chronic intestinal Candida (putatively caused by overgrowth of Candida albicans) has been cited as a possible contributor to ME/CFS [61,62] and is associated with several diseases of the gastrointestinal tract [63].

Prior to developing ME/CFS, the patient also reported being bitten by several ticks in Central America in her early 20s. However, she was never diagnosed or treated for Lyme disease. Untreated or inadequately treated Lyme disease can progress to a late disseminated disease after initial infection that can result in substantial disability [64,65]. Lyme disease can result in neurological manifestations [64,66], in addition to chronic fatigue [64,67]. By the time the patient was bitten by ticks, she had been subjected to years of female hormones and antibiotics (which were likely unnecessary), along with daily consumption of dairy (which was likely contributory to chronic sinusitis). Therefore, years of potentially harmful and misdirected treatments may have created an environment more susceptible to disease. Lyme disease tests are falsely negative in 40.5% of cases (accordingly to a recent meta-analysis) [68], which further highlights the challenges in diagnosing Lyme disease.

Research studies have shown that patients with ME/CFS have abnormalities of the central and autonomic nervous systems, metabolic dysfunctions, compromised immunity, and chronic infections [9,10,11]. However, the overseeing physician was unable to provide an active diagnosis throughout the duration of treatment due to limitations with available clinical testing. Recent research has shown that there are no significant differences between antibody/antigen serology tests against common viral and bacterial pathogens in patients with severe ME/CFS compared to healthy controls [69]. As such, the patient’s past medical history and suspected causes of ME/CFS were often used to guide treatment recommendations. For example, although the overseeing physician initially believed Mycoplasma pneumonia (which has been documented in patients with ME/CFS) [70] was the primary contributor to the patient’s illness, after 1.5 years of minimal progress on a Mycoplasma pneumonia focused protocol (dominantly antibiotics), the overseeing physician recommended herbs for the patient to try for other possible infections (including Lyme disease and Epstein–Barr virus). This more comprehensive herbal approach for 2.5 years, combined with non-invasive therapies (light, EFT, and EMDR), was successful in getting the patient back to graduate school after becoming bedridden with ME/CFS 4.5 years prior. However, it was not possible to determine whether the improvements observed were mediated by immunologic, antifungal, antiviral, antimicrobial, or neurological effects of various treatments vs. simply a spontaneous remission (or placebo effect) over time. Studies have shown that after a period of 15 months, spontaneous recovery from ME/CFS rarely occurs [71], highlighting that the patient’s progress was likely attributed to various aspects of treatment. 

It could be argued that a combination of herbs that helped disintegrate drug-resistant biofilms, strengthen immunity, enhance detoxification, and target a wide range of possible infections (including Mycoplasma pneumonia, Candida, Lyme disease, and Epstein–Barr virus) likely played a critical role in improving the patient’s symptoms over time. Although herbs were used throughout treatment, a more comprehensive approach was taken in the latter half of treatment after antibiotics were abandoned. In addition, non-invasive therapies (i.e., light, EFT, and EMDR) added during the latter part of treatment may have helped create an environment more conducive for healing. However, given that all changes to the patient’s protocol resulted in Jarisch–Herxheimer reactions (with severe hypotension being the most dangerous) and that no meaningful shifts in the patient’s laboratory results occurred over a 4+ year period, further complicated determining what was most (or least) effective. 

Patients with ME/CFS may have chronic infections that will increase the likelihood of paradoxical reactions to treatment (Jarisch–Herxheimer reaction) (Table 1). Throughout treatment (September 2013 to August 2017), the patient experienced paradoxical responses (Jarisch–Herxheimer reaction) to all forms of treatment, which were suggestive of unresolved chronic infections. Indeed, authors have emphasized that the Jarisch–Herxheimer reaction is a necessary adverse reaction for achieving a cure from various infections [17,18,19,20,21,22,23,24,25,26]. Although the Jarisch–Herxheimer reaction is generally a transient reaction, the patient experienced ongoing Jarisch–Herxheimer reactions that lasted for years. Any perturbation in her treatment protocol was often enough to reinstate a Jarisch–Herxheimer reaction. To manage Jarisch–Herxheimer reactions, the patient relied primarily on herbal recommendations from her overseeing physician for detoxification support. In addition, she frequently had to pause (or slow treatment), and daily light therapy was often crucial. 

It could be argued that the patient’s long-term struggles with Jarisch–Herxheimer reaction have been a necessary process for healing. However, care must be taken to distinguish the Jarisch–Herxheimer reaction (which can be beneficial and is not specific to the ME/CFS patient) from post-exertional malaise (which is the hallmark symptom of ME/CFS and detrimental to the health of the ME/CFS patient) (Table 1). Recent recommendations from the Mayo Clinic acknowledge that graded exercise therapy is contraindicated for patients with ME/CFS [13]. If a patient improves with graded exercise, he/she does not have post-exertional malaise, and thus he/she does not have ME/CFS. Graded exercise protocols have been shown to have detrimental effects on patients with ME/CFS due to mitochondrial dysfunctions, low oxygen update, abnormal autonomic responses, and immunological abnormalities to name a few [1,10,72]. Therefore, it is not surprising that the patient reported a decreased tolerance to exertion when under treatment. This sheds light on the overlap between the two responses (i.e., post-exertional malaise and Jarisch–Herxheimer reaction), as a temporary worsening of symptoms from treatment may require decreased exertion to avoid long-term setbacks. Excessive exertion can result in an irreversible decline in function (which has been reported by patients with severe ME/CFS) [2].

In summary, this case report documents the progression of a patient with ME/CFS over 4 years and her continuous paradoxical reactions to treatment (which we propose have been a necessary aspect for healing). Patients with ME/CFS tend to have severe sensitivities to treatment (as documented elsewhere [27] and in the current report), highlighting that potential therapies need to be performed with extreme care to avoid detrimental results. Given the patient’s case history (e.g., chronic sinusitis, amenorrhea, tick bites, congenital neutropenia, psychogenic polydipsia, food intolerances, and hypothyroidism), we hypothesize that these events contributed to her development of severe ME/CFS in her late 20s. Although the patient improved on a protocol combining herbs, traditional pharmaceuticals, and non-invasive therapies (LED colored lights, EFT, and EMDR), these treatments were experimental as the overseeing physician was unable to provide an active diagnosis (which was complicated by limitations with available clinical testing). From a clinical standpoint, this report aims to alert health care providers to the complications in treating patients with ME/CFS and provide possible guidance on future research and treatment options for ME/CFS. 

## Figures and Tables

**Table 1:** Jarisch–Herxheimer Reaction vs. Post-Exertional Malaise.

	Jarisch–Herxheimer Reaction [17,18,19,20,21,22,23,24,25,26]	Post-Exertional Malaise [1,12,13]
Definition	Worsening of existing symptoms (and appearance of new symptoms) following treatment in several infectious diseases (including viral, bacterial, and fungal). It should not be confused with a drug allergy or adverse reaction to treatment.	Worsening of existing symptoms following excessive cognitive, physical, orthostatic, emotional, or sensory challenges that were previously tolerated.
Onset	Typically occurs within 24 h but may be delayed by 7–14 days.	Typically occurs within 48 h of excessive exertion.
Duration	Hours or days	Hours, days, weeks, or months
Specific to ME/CFS	No	Yes
Controlling	Patients with ME/CFS may need to commence treatment with very low dosages and titrate upwards with caution [27].	Pacing (i.e., staying within energy envelope) is necessary to avoid.
Triggers	Should be expected by all patients receiving treatment (or related herbal treatment) for infectious diseases if an adequate infectious load is present.	Can be triggered by the most mundane activities (conversation and showering), depending on ME/CFS severity.
Example of Symptoms Experienced by Patient over Course of 4+ Years *	Severe hypotension (systolic blood pressure would frequently drop below 80 mm Hg) Severe musculoskeletal and joint pain (often uncontrollable) Migraines Sore throat Severe bloating and intestinal cramps Extreme fatigue (bedridden) Sweating and chills Nausea Brain Fog	Hypotension General body aches Headaches Sore throat Extreme fatigue (bedridden) Nausea Brain fog

* Symptoms of post-exertional malaise vs. Jarisch–Herxheimer reaction commonly overlap. If the patient was in a Jarisch–Herxheimer state and overexerted, then the Jarisch–Herxheimer reaction would simply worsen. The Jarisch–Herxheimer reaction is more commonly known as die-off.

**Table 2:** Serology Laboratory Tests Ordered at Initial Visit to Rheumatologist (August 2013) after ME/CFS Diagnosis.

ACL (Anti-Cardiolipin) Antibodies (IgM, IgG, IgA)
Ammonia
Amylase
ANA (Antinuclear Antibodies) with reflex to 11
Anti-CCP (Cyclic Citrullinated Peptide) lgG Semi-Quantitative
ASO (Antistreptolysin O) Antibodies
B2M (Beta 2 Microglobulin) Tumor Marker
Bilirubin, Direct
Chlamydia pneumonia (IgG/IgM)
Chlamydia trachomatis (IgM)
Complement C3a/C4a
CBC (Complete Blood Count) with Differential/Platelet
CK (Creatine Kinase)
Complete Metabolic Panel
CRP (C-Reactive Protein)
Ferritin
Free Kappa Light Chains
Free Lambda Light Chains
GGT (Gamma-Glutamyl Transferase)
G6PD (Glucose-6-phosphate dehydrogenase) Enzyme
Hemoglobin A1c
HNK-1 (Human Natural Killer-1) CD57
Immunoglobulins (IgA, IgG, IgM)
LDH (Lactic Acid Dehydrogenase)
Lipase
Lyme Western Blot
Magnesium
Mycoplasma pneumonia (IgG/IgM)
Natural Killer Cell Surface Antigen (CD56/16)
Phosphorus
Rheumatoid Factor
Rheumatoid Factors (IgM, IgG, IgA)
Sedimentation rate
Serum Iron
Serum Protein Electrophoresis
Thyroid Panel (TSH, T3, T4, Free T4)
Uric Acid
Urinalysis
Vitamin D (25-Hydroxy)

**Table 3:** Medications Prescribed during Phase 1 of Treatment (September 2013–February 2015).

Name	Reason Prescribed
Prescriptions	
Doxycycline	Antibiotic (Mycoplasma pneumonia)
Clarithromycin	Antibiotic (Mycoplasma pneumonia)
Azithromycin	Antibiotic (Mycoplasma pneumonia)
Dipyridamole [31]	Increase antibiotic potency
Nystatin	Antifungal
Fluconazole	Antifungal
Gentamycin	Nebulized antibiotic (Mycoplasma pneumonia)
Glutathione	Nebulized antioxidant for detoxification
Hydroxychloroquine	Pain and inflammation; increase antibiotic potency
Fludrocortisone	Raise aldosterone to improve hypotension
Armour Thyroid	Hypothyroidism
Levothyroxine	Hypothyroidism
Supplements	
Succinic Acid	Detoxification
N-Acetyl Cysteine	Detoxification; biofilm disruptor
Liver Detox Blend ^†^	Detoxification
Modified Citrus Pectin	Detoxification
Bromelain	Pain and inflammation; biofilm disruptor
Boswellia/Curcumin	Pain and inflammation
Colostrum	Immune
Cordyceps	Immune
Kelp (iodine)	Immune and thyroid
Ashwagandha	Adaptogen
Rhodiola Extract	Adaptogen
Eleutherococcus	Adaptogen
Licorice Root	Raise aldosterone to improve hypotension
Phosphatidyl Serine	Brain fog
Artemisian	Antimicrobial
Berberine	Antifungal
Silver Hydrosol	Antimicrobial (Mycoplasma pneumonia)
Olive Leaf Extract	Antimicrobial (Mycoplasma pneumonia)
Anantamul	Antimicrobial (Mycoplasma pneumonia)

Prescribing physician MD, PhD. All oral unless otherwise indicated. Patient was also taking several supplements on her own that the physician approved (e.g., probiotics, zinc, vitamin D, and fish oil). ^†^ Wide range of herbs that includes milk thistle, schisandra, bupleurum, dandelion, scute, N-acetyl cysteine, methionine, barberry, turmeric and more.

**Table 4:** Medications Prescribed during Phase 2 of Treatment (February 2015–August 2017).

Name	Reason Prescribed
Prescriptions	
Armour Thyroid	Hypothyroidism
Levothyroxine	Hypothyroidism
Cholestyramine	Detoxification
Supplements	
Cat’s Claw (Uncaria Tomentosa)	Antimicrobial (Lyme disease)
Neem	Broad-Spectrum Antimicrobial
Inosine	Antiviral (Epstein–Barr virus)
PABA (Para-aminobenzoic Acid)	Antiviral (Epstein–Barr virus)
DMAE (Dimethylaminoethanol)	Antiviral (Epstein–Barr virus)
L-Lysine	Antiviral (Herpes simplex virus)
Drynaria	Osteopenia
Bamboo Extract	Osteopenia
Andrographis	Immune
Astragalus	Immune
Iporuru	Pain and inflammation
Zeobind	Heavy Metal Chelation
Modified Citrus Pectin	Heavy Metal Chelation

Prescribing physician MD, PhD. All oral unless otherwise indicated. Patient was also taking several supplements on her own that the physician approved (e.g., probiotics, zinc, vitamin D, and fish oil), in addition to several supplements shown in Table 3.

**Table 5:** Summary of Treatments Used by Patient Over 4+ Year Period.

	Phase 1	Phase 2
	(September 2013–February 2015)	(February 2015–August 2017)
Long-Term Antibiotics ^1^	x	
Long-Term Antifungals ^1^	x	
Long-Term Thyroid Medication ^1^	x	x
Miscellaneous Prescriptions ^1^	x	x
Very Restricted Specialized Diet for Seriously Ill ^2^	x	x
Herbs ^1^	x	x
Eye Movement Desensitization and Reprocessing (EMDR) ^3^		x
Emotional Freedom Techniques (EFT) ^3^		x
Light Therapy ^3,4^		x

^1^ Prescribed by MD, PhD (overseeing physician). Miscellaneous prescriptions included Dipyridamole, Nebulized Glutathione, Hydroxychloroquine, Fludrocortisone, and Cholestyramine. ^2^ Based on recommendations from the book “Food is Your Best Medicine”, by Henry Bieler, MD [14]. Started immediately when health seriously declined, several months before initiation of formal treatments. ^3^ Core treatment during sessions with PhD clinical psychologist. ^4^ Light therapy was regularly performed at home (up to 6 h/day). In time, this was incorporated with EFT and EMDR.

## Data Availability

Not applicable.
